# Measurement of electrostatic tip–sample interactions by time-domain Kelvin probe force microscopy

**DOI:** 10.3762/bjnano.11.76

**Published:** 2020-06-15

**Authors:** Christian Ritz, Tino Wagner, Andreas Stemmer

**Affiliations:** 1 Nanotechnology Group, ETH Zürich, Säumerstrasse 4, 8803 Rüschlikon, Switzerland; 2 present address: Zurich Instruments AG, Technoparkstrasse 1, 8005 Zürich, Switzerland

**Keywords:** atomic force microscopy (AFM), electrostatic height error, extended Kalman filter, Kelvin probe force microscopy (KFM), time domain

## Abstract

Kelvin probe force microscopy is a scanning probe technique used to quantify the local electrostatic potential of a surface. In common implementations, the bias voltage between the tip and the sample is modulated. The resulting electrostatic force or force gradient is detected via lock-in techniques and canceled by adjusting the dc component of the tip–sample bias. This allows for an electrostatic characterization and simultaneously minimizes the electrostatic influence onto the topography measurement. However, a static contribution due to the bias modulation itself remains uncompensated, which can induce topographic height errors. Here, we demonstrate an alternative approach to find the surface potential without lock-in detection. Our method operates directly on the frequency-shift signal measured in frequency-modulated atomic force microscopy and continuously estimates the electrostatic influence due to the applied voltage modulation. This results in a continuous measurement of the local surface potential, the capacitance gradient, and the frequency shift induced by surface topography. In contrast to conventional techniques, the detection of the topography-induced frequency shift enables the compensation of all electrostatic influences, including the component arising from the bias modulation. This constitutes an important improvement over conventional techniques and paves the way for more reliable and accurate measurements of electrostatics and topography.

## Introduction

Electrostatic forces are important interactions in non-contact atomic force microscopy (NC-AFM). They arise from differences in the work function of the tip and the sample, from trapped charges, or from potentials applied to active nanoelectronic devices. Kelvin probe force microscopy (KFM) is a technique used to quantitatively characterize such electrical properties [1–3]. It is applied to map material compositions via changes in the work function, to localize charge distributions in dielectric samples [4–5], and to characterize doping profiles via scanning capacitance measurements [6]. Especially in the field of nanoelectronic devices, this kind of electrical characterizations is of great interest. Local potential drops across active nanostructures reveal information about the local resistivity and can provide crucial insights into the operation mode [7–8].

The principle of KFM is based on modulating the electrostatic force or force gradient. To this end, the tip–sample bias voltage is set to

[1]
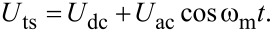


When modeling the tip–sample system as a capacitance, the resulting electrostatic force is defined as

[2]
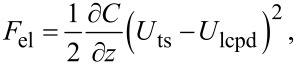


where C is the tip–sample capacitance and *U*_lcpd_ is the local contact potential difference. *U*_lcpd_ contains information about both the contact potential difference and the potential arising from charge interactions [9–10]. Consequently, the electrostatic force gradient is given by

[3]
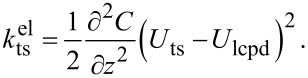


It should be noted that capacitance C can be a function of not only the distance *z* but also of the applied bias voltage *U*_ts_. This should be observed especially at large bias in conjunction with a semiconducting tip or sample due to band bending [11]. In this case, the model can be extended by considering the Taylor expansion of the capacitance with respect to the tip–sample bias, defined by

[4]
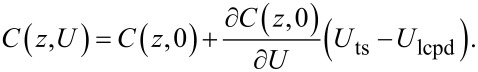


Different spectral components arise due to the electric modulation at ω_m_. Namely, a static component (dc) as well as components at the modulation frequency ω_m_ and at the second harmonic frequency 2ω_m_. These spectral components are defined by

[5]
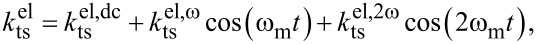


with

[6]
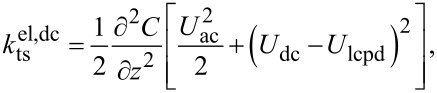


[7]
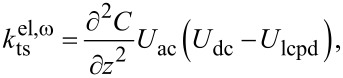


and

[8]
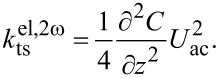


In conventional frequency-modulated (FM-) KFM, the contributions at ω_m_ and 2ω_m_ are detected via lock-in techniques, either at the Δ*f* output of a phase-locked loop (PLL) [12] or by detecting the sidebands of the cantilever oscillation [13].

In closed-loop FM-KFM, a feedback loop is employed to nullify the component at ω_m_ by adjusting *U*_dc_. The surface potential is then found as *U*_lcpd_ = *U*_dc_. The response at the second harmonic contains additional information about the capacitance gradient C^′′^ = ∂^2^C/∂*z*^2^. This signal is interesting in itself as it contains information about both geometric and electronic properties of tip and sample, e.g., the dielectric properties of a sample or the quantum capacitance [14]. Furthermore, this signal can be used to adjust the sensitivity of the KFM feedback loop [15].

Open-loop KFM techniques exploit the relationship of the contributions at ω_m_ and 2ω_m_. Namely, *U*_lcpd_ can be found from their ratio 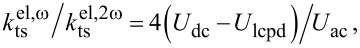 which otherwise only depends on the parameters of the applied modulation, *U*_ac_ and *U*_dc_. When applying KFM as an open-loop technique, optimization of filter parameters is possible, leading to an improved controller performance [16].

For correct height measurements, it is necessary to compensate the electrostatic forces [17–19]. With a closed KFM loop, these forces are minimized by definition. However, a static contribution, which is defined by

[9]
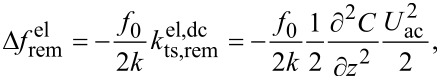


remains uncompensated. This component results from the bias modulation itself and influences the topography measurement. The effect is amplified when a significant change in the capacitance gradient is present. To reduce the impact of this component onto the height measurement, the modulation amplitude *U*_ac_ must be minimized. Since the signal-to-noise ratio (SNR) scales with *U*_ac_, this is only possible to a certain extent. Another possibility for compensating the remaining frequency shift is the use of two-pass methods with feed-forward compensation techniques [20–21].

In this paper, we present a time-domain (TD) controller for KFM as a single-pass solution to the problem outlined above. Our method uses a Kalman filter as a state observer to continuously recover the full Δ*f*(*U*_ts_) parabola, also named Kelvin parabola. The maximum frequency shift Δ*f*_topo_, the contact potential difference *U*_lcpd_, and the capacitance gradient C^′′^ are evaluated in real time.

When applied as closed-loop technique, the height feedback can be performed on Δ*f*_topo_, where all electrostatic forces are compensated, including the static contribution 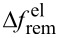. So far, recovering and fitting the Kelvin parabola is known as an open-loop technique, the so-called Kelvin probe force spectroscopy [22–25]. A real-time closed-loop technique has not been reported yet.

Furthermore, our method automatically determines the estimated error signals and the signal correlation coefficients. The error signals of the sample properties contain an evaluation of the accuracy at each position, which depends on the present surface properties due to the nonlinearity of the system. The signal correlation coefficients contain information about coupling and crosstalk between the channels estimated. Our method can be applied in closed-loop as well as in open-loop mode. Results of both modes are discussed in this paper.

## Detection Principle

The overall frequency shift in FM-AFM can be separated into a component induced by surface topography, Δ*f*_topo_, and a component induced electrically, Δ*f*_el_, therefore

[10]
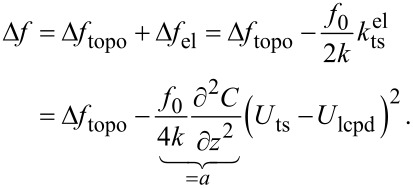


The coefficient *a* is proportional to the capacitance gradient C^′′^ and has the unit of Hz V^−2^. It is one of the three sample properties that are continuously estimated by the controller. The additional two estimated properties are Δ*f*_topo_ and the surface potential *U*_lcpd_.

**Figure 1:**
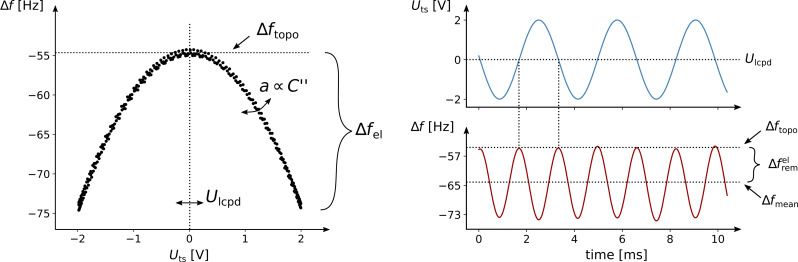
A modulation of the tip–sample bias *U*_ts_ leads to a response in the frequency shift Δ*f*. The time-resolved measurements of both signals are shown on the right, the resulting Kelvin parabola on the left. From this information, the controller continuously estimates the contact potential difference *U*_lcpd_, the capacitance gradient C^′′^, and the frequency shift due to the topography alone, Δ*f*_topo_. Sample: Single-layer graphene on SiO_2_; Tip: Olympus AC240TM-R3.

By applying a sinusoidal modulation to the tip–sample bias *U*_ts_, the parabolic dependence in Equation 10 can be observed. An example of the resulting Kelvin parabola is shown in Figure 1. The data was obtained above a single-layer graphene flake. The figure shows the influence of the three sample properties on the shape of the parabola and gives an overview of the different components of the frequency shift Δ*f*.

Traditional closed-loop controllers for KFM use lock-in techniques to measure the response of the cantilever at the modulation frequency ω_m_ (not visible in Figure 1 since *U*_dc_ ≈ *U*_lcpd_) and at 2ω_m_ [13,26–27]. A feedback loop is used to adjust *U*_dc_ in order to nullify the component at ω_m_. If this is achieved, the resulting Δ*f*_mean_, which is used for the topography control, is independent of the surface potential. However, it is still affected by the static contribution Δ*f*_rem_. As can be seen in Equation 9, this component depends on C^′′^ and may change across material borders, inducing a topographic height error.

The approach discussed in this paper tracks the time-resolved Δ*f*-response to the applied bias voltage. A state observer based on an extended Kalman filter is used to continuously fit the resulting parabola. The output of the time-based controller is an estimation of the topography-induced frequency shift Δ*f*_topo_ (which is not affected by Δ*f*_rem_), the surface potential *U*_lcpd_, and the coefficient *a*. Since our technique is based on the time domain, it is not limited by the bandwidth of additional filters in the loop, for example lock-in amplifiers. Therefore, we expect to achieve higher bandwidths compared to standard KFM implementations.

An asymmetrical bias dependence, as caused by band bending, may be included. When considering the Taylor expansion in Equation 4, the overall frequency shift results in

[11]
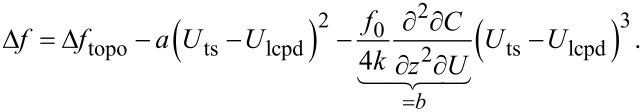


The sample property *b* is introduced to the estimator as an additional state. This gives the controller the ability to locally fit a third-order parabola to the KFM data. Similarly, other sources of asymmetry may be included here, although the detectability of the system needs to be confirmed. For instance, the polarizability of a sample could be added through an additional state γ and by modifying Equation 10, e.g., according to Equation 8 in [28].

For the sake of simplicity, this publication focusses on the implementation of the most basic case with three states, where only the quadratic influence on the Kelvin parabola is considered. The model of the measurement system, which is used by the state observer, has to include the dynamics of the detection system. If a PLL is used, its transfer function, which is known for a given cantilever and given PI gains, can be approximated by the transfer function of a low-pass filter.

Using the relationship shown in Equation 10 and the transfer function of the detection system, the sample properties, the expected error signals, and the correlation coefficients can be determined from the bias dependence of the frequency shift Δ*f*(*U*_ts_). For robust estimation, it is beneficial if the surface potential *U*_lcpd_ is within the modulated bias voltage *U*_ts_(*t*). A single-sided modulation on one branch is feasible but not recommended.

## Controller Design

**Figure 2:**
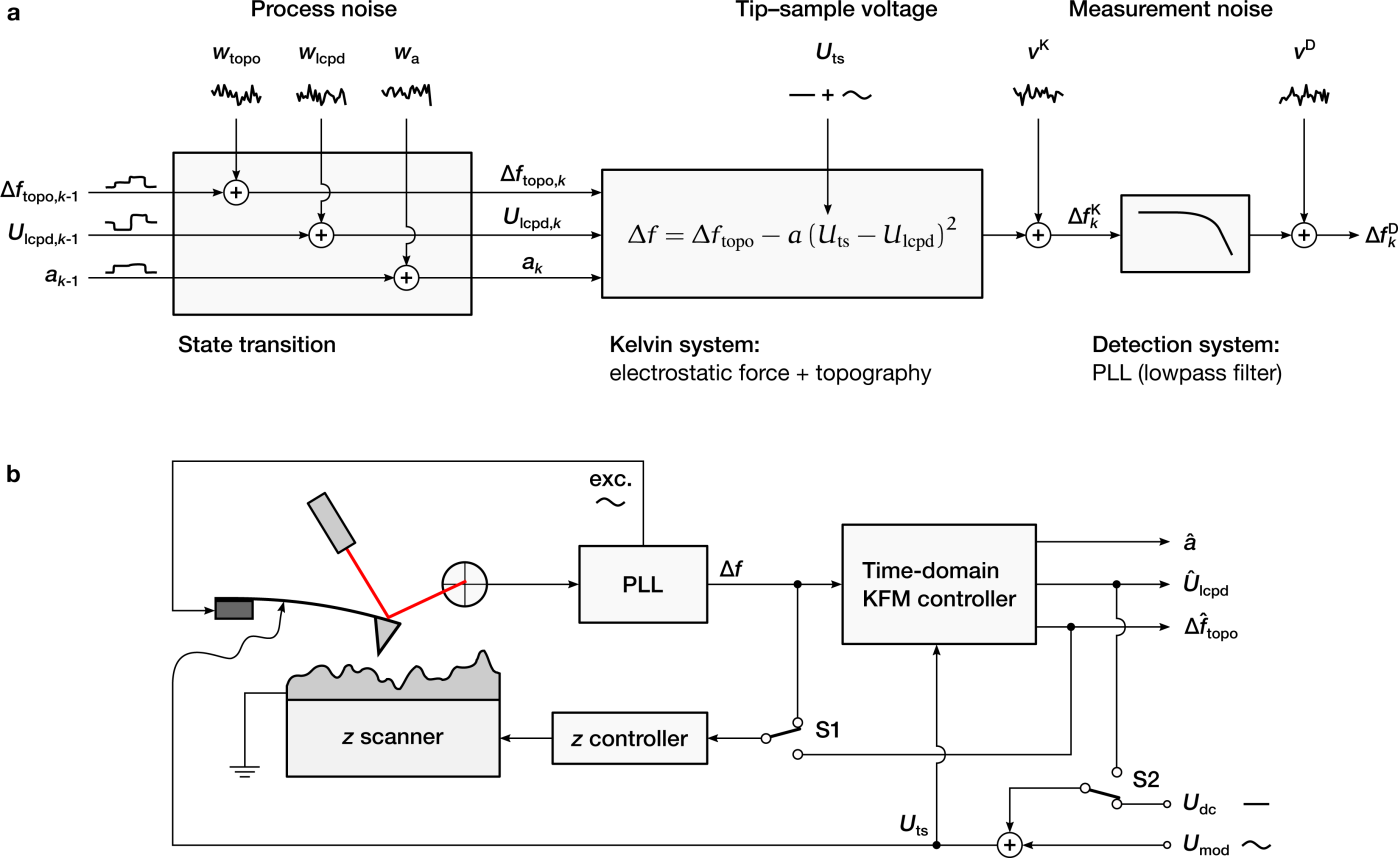
Design and operation of the time-domain KFM controller. (a) Underlying system model. (b) Measurement setup. The estimated topography-induced frequency shift, 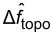, can be used in place of Δ*f* to minimize electrostatic height errors (switch S1). The electrostatic force gradient between tip and sample is minimized by adding 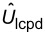 as a dc bias to *U*_ts_ (switch S2).

The state-space representation of the Kelvin system and the detection system are derived separately, using the superscripts K and D, respectively. The two subsystems will be merged in Equations 22–26. An illustration of the two subsystems in the overall KFM model is shown in Figure 2a.

The Kelvin system, i.e., the measurement system without the detector, has the state vector

[12]
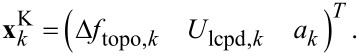


Index *k* is the discrete sampling applied, *t_k_* = *k*Δ*t*. The model assumes that the sample properties remain constant between two discrete measurements, thus

[13]
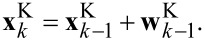


Deviations from this assumption, for example due to scan movement, are introduced by the vector

[14]
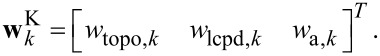


This vector, the so-called transition noise vector, is anticipated to be Gaussian white noise 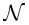
(0,**Q**^K^) with the covariance matrix

[15]
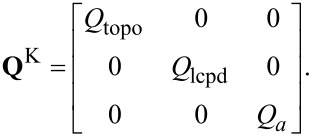


The output of the Kelvin system is the scalar value of the intrinsic frequency shift 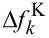. It is defined by

[16]
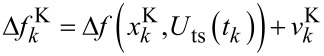


[17]
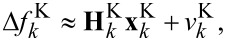


using the definition in Equation 10. The measurement noise 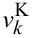 is assumed to be Gaussian white noise and is defined by 
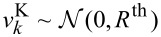
. This is the case for measurements where thermal noise is the dominating noise source, i.e., when the modulation frequency is below the crossover of thermal white noise and (with increasing frequency) detection noise [29]. The power spectral density (PSD) of the thermal noise can be calculated as

[18]
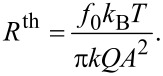


For detection-noise-limited measurements, an additional state has to be introduced to keep track of the dynamics of the apparent noise.

The approximation in Equation 17 contains the system output matrix 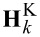. It depends on the current tip–sample voltage *U*_ts_*_,k_* and linearizes the system around the state 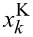. It is defined by

[19]
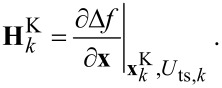


The intrinsic frequency shift, 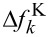, then enters the detection system, which is modeled by the linear time-invariant system represented by the state transition matrix **F**^D^, input matrix **G**^D^, output matrix **H**^D^, and feed-through coefficient *D*^D^:

[20]
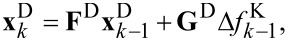


[21]
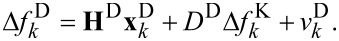


The observed frequency shift Δ*f_k_* corresponds to the output of the detection system, 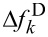. If a PLL is used as a detection technique, the above system can be approximated by a low-pass filter. The noise introduced at the output of the detection system is modeled by Gaussian white noise with 
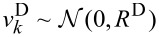
.

Merging the two subsystems leads to the state-space representation given by

[22]
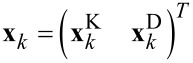


[23]
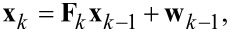


[24]
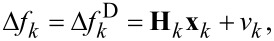


with

[25]
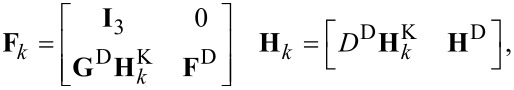


and the transition and observation noise

[26]
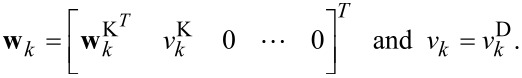


The state observer uses the derived state-space description of the measurement system for continuously evaluating *x*^K^, i.e., the sample properties, according to the measured frequency shift. Due to the nonlinearity of the system, nonlinear state estimation is required. The extended Kalman filter (EKF) is a straightforward and computationally efficient method for dealing with such systems. The implementation shown uses the notation of Simon [30].

The algorithm starts with an arbitrary initialization of the a posteriori state estimate 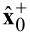 and the initial state covariance matrix 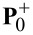 (e.g., 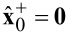 and 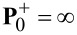). Afterwards, two steps have to be applied for each iteration *k*. The prediction step calculates the a priori estimate 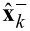 from the previous a posteriori estimate 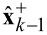 according to

[27]
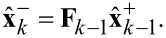


Note that **F***_k−_*_1_ contains the linearization through the output matrix 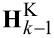 and has to be updated beforehand. The linearization step shown in Equation 19 is performed around the last estimate 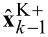 and the known bias voltage *U*_ts_*_,k−_*_1_. Similarly, the system a priori covariance matrix 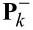 is calculated from 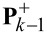 as in

[28]
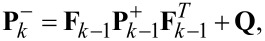


with the transition noise matrix

[29]
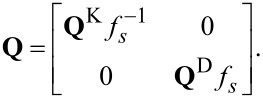


The transition noise matrix of the detection system **Q**^D^ is the zero matrix with the PSD of the thermal noise *R*^th^ at its top-left position. The covariance matrix **Q**^K^ contains the assumed PSD of the sample properties. The values of **Q**^K^ are design parameters and determine how much change may be expected between two sampled points. For the TD controller, these values represent the gain of each estimated channel. The sampling-rate-independent continuous noise density **Q**^K^ (**Q**^D^) is divided (multiplied) by *f_s_* to obtain the discrete noise density **Q**.

After the prediction step, a measurement update is performed. The Kalman gain **K***_k_* is calculated as

[30]
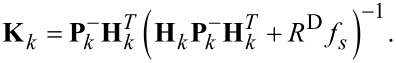


It determines how much weight should be put onto the currently measured Δ*f_k_*. Again, **H***_k_* has to be updated before using Equation 19 where the linearization is made around the time-updated state 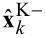 and the current bias voltage *U*_ts_*_,k_*.

The a posteriori estimate 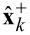 and covariance 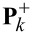 are then calculated as

[31]
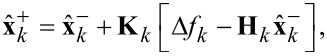


[32]
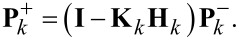


The a posteriori values are considered to be the best estimates at time *k*. During the estimation, a negative value for the coefficient *a* may result. Due to the definition in Equation 10, this is physically not valid and can be corrected by projecting state 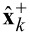 back to the allowed region [31].

The state covariance matrix 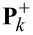 provides additional information about the measurement. The estimated variances σ*_i,k_* of state **x***_k_* are the diagonal values of 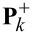 and represent the expected error signals. These values have to be interpreted carefully since they depend on the design parameters in **Q**^K^. A small value for *Q_i_* is interpreted as an increased confidence towards the prediction of this channel resulting in a smaller variance. However, due to the nonlinearity of the measurement system, the error signals also depend on the estimated sample properties. This allows one to distinguish regions where an increased SNR can be expected from regions where the controller is less confident due to a smaller SNR.

Furthermore, the correlation coefficients can be calculated as

[33]
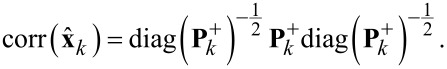


They contain information about coupling and crosstalk between the different states. If the magnitude of a correlation coefficient is close to one, crosstalk between these two channels has to be expected, while a value close to zero means little to no crosstalk.

The measurement setup is shown in Figure 2b. The estimator can be applied in both open-loop and closed-loop mode. When using 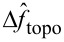 as control signal for the topography measurement (switch S1), the controller aims to nullify the electrostatic height error. The electrostatic force between tip and sample is minimized when adding 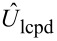 as dc bias to *U*_ts_ (switch S2). If the topography control uses 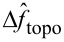 as feedback signal, the electrostatic forces are compensated already, even for an open KFM loop. Nevertheless, closing the KFM loop prevents a single-sided modulation where the controller is much less sensitive and may lose its stability.

To initially tune the closed-loop controller, it helped to approach the sample with Δ*f* as feedback parameter and an open KFM loop (open S1 and S2). After approaching the surface, the input signals Δ*f* and *U*_ts_ of the estimator and the reconstructed surface properties 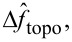 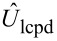 and 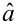 were observed on an external oscilloscope. The controller could be stabilized by adjusting the values of the transition noise matrix **Q**^K^. After the output signals of the estimator were steady, both switches were closed. Note that the topography setpoint should be adjusted before closing S1 because Δ*f*_topo_ ≥ Δ*f*_mean_ as illustrated in Figure 1.

## Results and Discussion

The performance of the TD-KFM controller is demonstrated on multilayer graphene samples. The samples were obtained by exfoliation from bulk graphite [32–33] and deposited on a piece of Si/SiO_2_. Measurements were carried out using an Asylum Research Cypher AFM connected to a Zurich Instruments HF2 lock-in amplifier.

**Figure 3:**
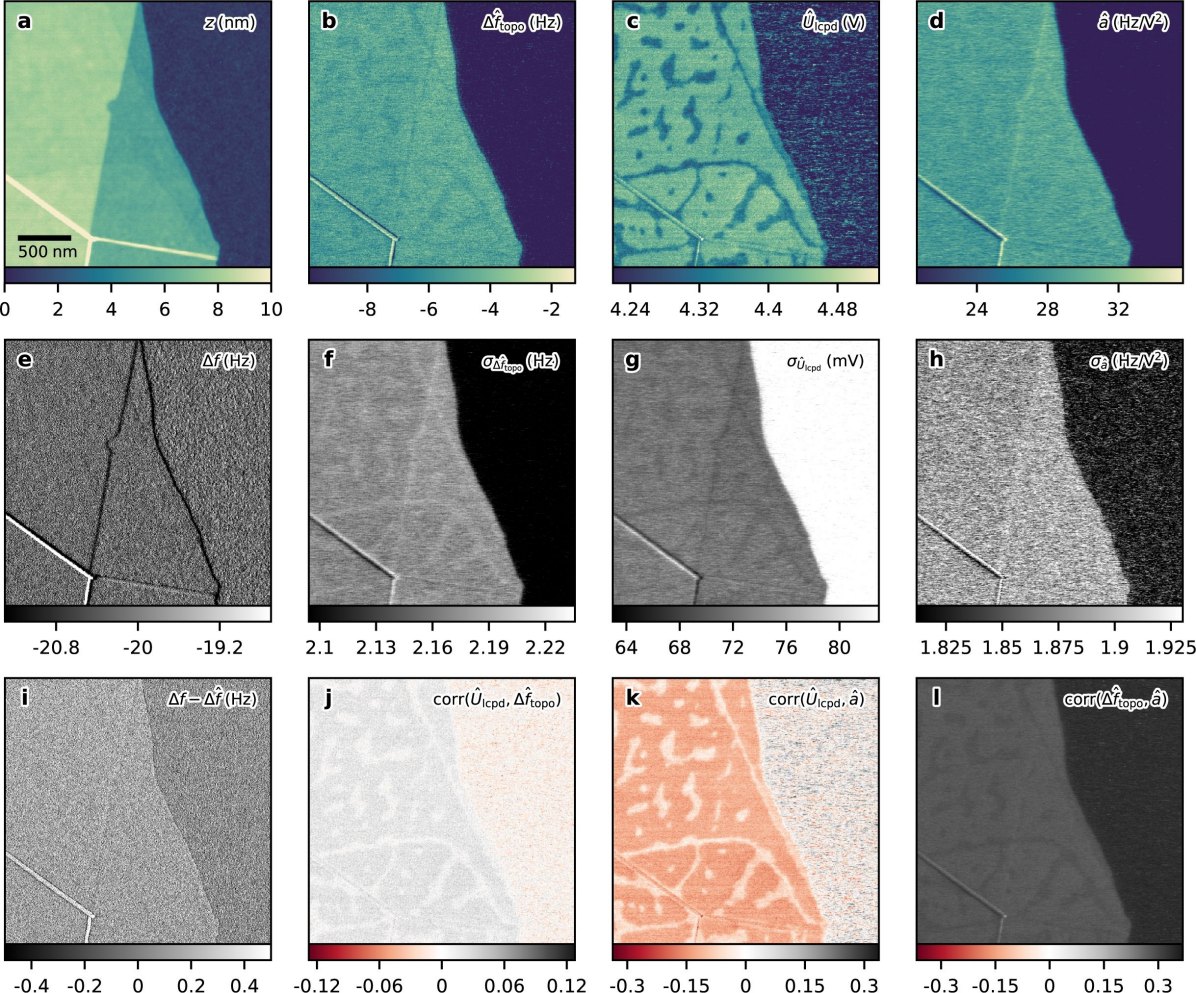
Open-loop TD-KFM of a few-layer graphene flake. (a) Topography, (b) estimated topography-induced frequency shift 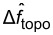, (c) estimated surface potential 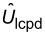, and (d) estimated coefficient 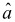. (e) The frequency shift Δ*f* was kept constant at −20 Hz by the topography feedback. (f–h) and (j–l) show the variance and mutual correlations of the estimated states, respectively. (i) Residual 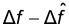 of the estimation. Parameters: *U*_ac_ = 1 V, *U*_dc_ = 4.3 V, *f*_m_ = 500 Hz, *f*_s_ = 3.6 kHz, PLL bandwidth: 1.5 kHz, *R*^th^ = 1.55 × 10^−3^ Hz^2^ Hz^−1^, *Q*_topo_ = 4800 Hz^2^ Hz, *Q*_lcpd_ = 10 V^2^ Hz, *Q*_a_ = 400 Hz^2^ V^−4^ Hz; Detection noise *R*^D^ ≪ *R*^th^ is neglected here. Tip: Olympus AC240TM-R3, *A* = 14 nm, *k* = 2.3 N m^−1^, *f*_0_ = 71.04 kHz, *Q* = 134, *v*_scan_ = 2.5 μm s^−1^.

Figure 3 shows the results of the open-loop controller. During the FM-AFM measurement the tip voltage was modulated with an amplitude of *U*_ac_ = 1 V at *f*_m_ = 500 Hz. The frequency shift Δ*f* was obtained by using a PLL with a bandwidth set to 1.5 kHz, such that the electrostatic contributions at *f*_m_ and 2*f*_m_ were detected. Both tip voltage and frequency shift were recorded at a sampling rate of *f*_s_ = 3.6 kHz.

The surface topography is shown in Figure 3a, the obtained frequency shift is shown in Figure 3e. During postprocessing, the recorded Δ*f* signal and bias voltage are fed to the TD-KFM controller. From the hidden contributions at *f*_m_ and 2*f*_m_, the state observer reconstructed the sample properties, which are shown in Figure 3b–d. The topography-induced frequency shift Δ*f*_topo_ in Figure 3b is far from the −20 Hz Δ*f* setpoint used for the height feedback, indicating a large influence of the electrostatic force gradient. The material contrast present can be explained partially by differences in the surface potential, which was not compensated during this measurement, and partially by differing contributions due to 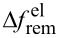. This contribution causes the bias-induced height error and will be further discussed below for the closed-loop demonstration. The estimate of the surface potential, depicted in Figure 3c, reveals the electrostatic potential above the graphene flake and the SiO_2_ substrate. The patterns observable on the graphene flake are most likely caused by water droplets, which have formed due to the ambient conditions [34–35]. Small changes in the patterns were observed between two different scans (roughly 20 minutes apart) due to adsorption and desorption. The estimate of coefficient *a* is depicted in Figure 3d, showing a material contrast caused by the difference in the capacitance gradient. The expected state variances are shown in Figure 3f–h. Due to the nonlinearity of the KFM system, the uncertainty of an estimated channel depends on the present state vector. A larger value for coefficient *a*, for example, increases the SNR of the surface potential measurement, leading to a smaller variance. A larger confidence is then attributed to the *U*_lcpd_ channel. Consequently, the expected error of the surface potential above the graphene flake is smaller than that above the SiO_2_ substrate. The residual of the state observer 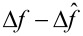 is shown in Figure 3i. This signal contains the amount of the frequency shift that could not be attributed to any surface property, for example the estimated measurement noise. In the present case, a material-dependent offset is visible. A mean value of 0.15 Hz can be extracted above the graphene flake while an average value of 0.07 Hz is found above the SiO_2_ substrate. This material-dependent component indicates the presence of coefficient *b*, which was approximated to be zero in the model assumptions. The presence and the magnitude of coefficient *b* are discussed in Supporting Information File 1, Figure S1. Figure 3j–l shows the correlation coefficients of the sample properties.

**Figure 4:**
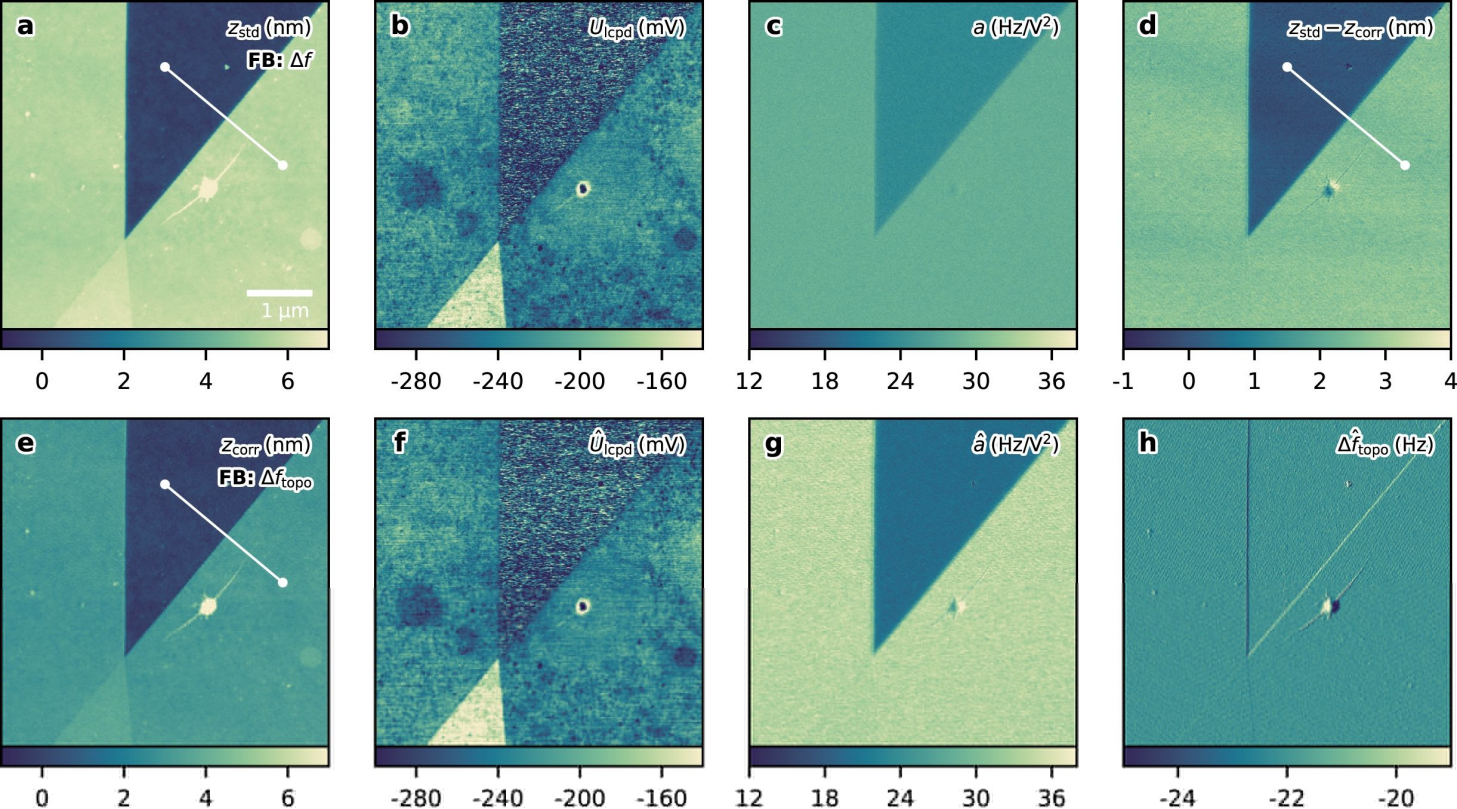
Comparison of standard single-scan FM-KFM and closed-loop TD-KFM of two overlapping graphene flakes. (a–c) Topography, *U*_lcpd_, and coefficient *a* obtained from a FM-KFM scan with direct sideband detection. (e–h) Topography, 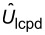, coefficient 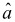, and 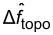 obtained by TD-KFM. The estimated topography-induced frequency shift 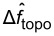 was used as topography feedback here. (d) Height difference between the TD-KFM and standard FM-KFM scan. The cross sections indicated by the white lines are shown below in Figure 5. Parameters FM-KFM: Δ*f*^set^ = −65 Hz, *U*_ac_ = 2 V, *f*_m_ = 1.5 kHz, PLL bandwidth: 500 Hz; TD-KFM: 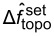 = −22 Hz, *U*_ac_ = 2 V, *f*_m_ = 253 Hz, *f*_s_ = 3.6 kHz, PLL bandwidth: 1 kHz, *R*^th^ = 1.66 × 10^−3^ Hz^2^ Hz^−1^, *Q*_topo_ = 5 Hz^2^ Hz, *Q*_lcpd_ = 0.004 V^2^ Hz, *Q*_a_ = 5 Hz^2^ V^−4^ Hz; Detection noise *R*^D^ ≪ *R*^th^ is neglected here. Tip: Olympus AC240TM-R3, *f*_0_ = 52.0 kHz, *k* = 1.0 N m^−1^, *Q* = 77.2, *A* = 12 nm (active ACL), *v*_scan_ = 1 μm s^−1^.

In the following, we demonstrate the application of the TD controller as a closed-loop technique. The sample examined consists of two overlapping single-layer graphene sheets, forming a bilayer in the lower part of the image. For comparing the TD controller to state-of-the-art KFM, the graphene flake was initially scanned by standard FM-KFM using sideband demodulation. A Kalman filter was used for optimal feedback control during this measurement [13]. The results of the standard technique are shown in Figure 4a–c. A second measurement was carried out using the TD-KFM controller. The topography-induced frequency shift 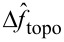 was used as control signal for the height feedback in this case. The results obtained from the state observer are depicted in Figure 4e–h.

The topography measurement of the standard KFM technique is influenced by the static contribution 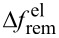. The contribution is proportional to the coefficient *a* and, thus, changes across the border of the graphene flake. The larger capacitance gradient present in the region of the graphene flake (see Figure 4c and Figure 4g) causes a more negative total frequency shift, which leads to an additional retraction of the tip. Consequently, an exaggerated height is measured. The TD controller is not affected by this artifact.

The difference between the two topography measurements is shown in Figure 4d. The height difference caused by the presence of 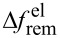 is roughly 2.6 nm, which is a large portion of the height measured by standard FM-KFM. This points out the importance of compensating the additional static contribution 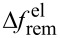 induced by the electric modulation.

**Figure 5:**
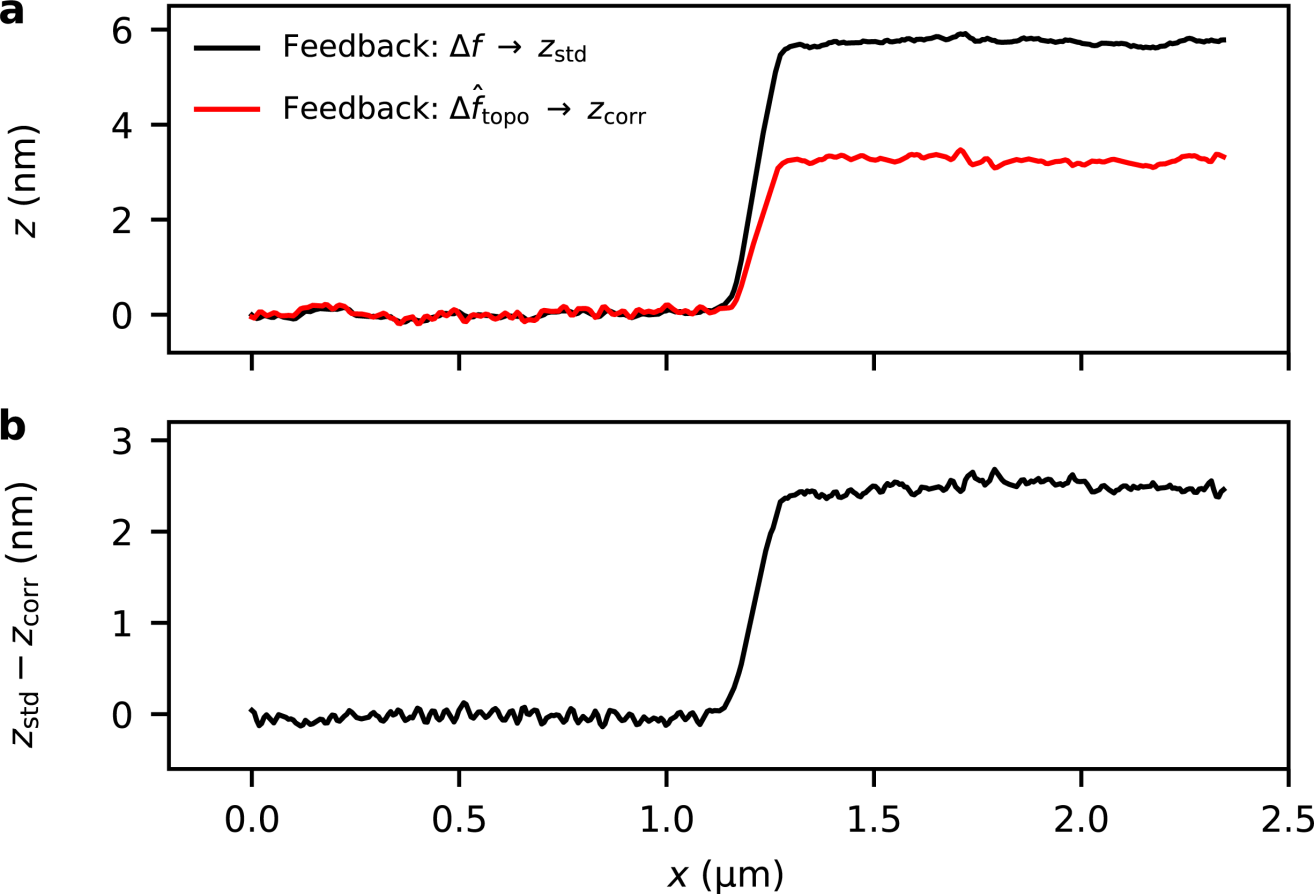
(a) Cross sections of the topography along the sections indicated in Figure 4 for single-scan FM-KFM (feedback on Δ*f*) and closed-loop TD-KFM (feedback on 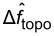). (b) Difference between both topography measurements. Closed-loop TD-KFM minimizes the electrostatic height error beyond what is achievable in standard FM-KFM.

The height profiles shown in Figure 5 are extracted from the topography images across the border of the graphene flake, as is indicated in Figure 4. Figure 5a shows the measured topography for both techniques and Figure 5b shows the obtained difference.

Standard FM-KFM and TD-KFM found similar values for the surface potential *U*_lcpd_. In both cases, the resolution depends on the controller settings and the same physical limitations apply. The results of the surface potential measurements are shown in Figure 4b and Figure 4f. The potential difference between the single- and the bilayer graphene is roughly 70 mV, which is similar to previously reported values [36].

The measurements of coefficient *a* are slightly different for the two techniques. It has to be noted, that the capacitance gradient is largely influenced by the tip–sample distance. The average tip–sample separation during the measurement with the standard KFM technique was larger above the graphene flake and smaller above the SiO_2_ substrate when compared to the TD measurement. This led to an increased value for *a* above the SiO_2_ substrate and a decreased value above the graphene flake.

## Conclusion

We demonstrated that surface topography and electrostatic properties can be directly estimated and separated from the frequency-shift signal. The state-space representation of FM-KFM was derived and an extended Kalman filter was introduced as a state observer. The estimation uses a linearization around the output matrix of the Kelvin system.

In our method, the maximum frequency shift is detected in real time and can be used as control signal for the topography feedback. The maximum frequency shift, Δ*f*_topo_, is not affected by the electrostatic force gradient, and therefore allows for the compensation of all electrostatic forces, including the remaining static contribution present in standard FM-KFM. The improved topography measurement was demonstrated by a closed-loop scan on a graphene flake.

The TD controller is also applicable as an open-loop technique. By modulating the tip–sample bias voltage during a regular FM-AFM measurement and by recording the frequency-shift signal and the applied bias voltage, the surface potential image can be reconstructed by the state observer. This is a very convenient way of performing KFM measurements by postprocessing. Furthermore, the dynamics of the controller can be studied and tested before conducting an actual closed-loop measurement.

The algorithm is reliable and robust for a wide range of gain values. The three parameters of the state transition matrix are the only tuning parameters needed, apart from the PSD of the thermal noise, which can be calculated beforehand.

## Code Availability

The code for the TD controller is made available upon request. It includes an open-loop version for postprocessing and a version for closed-loop application designed for the real-time unit (RTK) of the HF2 lock-in amplifier of Zurich Instruments.

## Supporting Information

File 1 Evaluation of the third sideband of the measurement shown in Figure 3.
