# Supplementary material for: Measurement of electrostatic tip–sample interactions by time-domain Kelvin probe force microscopy
Source: Beilstein J Nanotechnol. 2020 Jun 15;11:911–21. doi: 10.3762/bjnano.11.76 (PMC7308609; doi:10.3762/bjnano.11.76)
Supplement: File 1 — Evaluation of the third sideband of the measurement shown in Figure 3. [file Beilstein_J_Nanotechnol-11-911-s001.pdf]

BEILSTEIN JOURNAL OF NANOTECHNOLOGY

Supporting Information

for

Measurement of electrostatic tip–sample interactions by time-domain Kelvin probe force microscopy

Christian Ritz, Tino Wagner and Andreas Stemmer

Beilstein J. Nanotechnol. **2020,** *11,* 911–921. doi:10.3762/bjnano.11.76

Evaluation of the third sideband of the measurement shown in Figure 3

License and Terms: This is a supporting information file under the terms of the Creative Commons Attribution License (http://creativecommons.org/licenses/by/4.0). Please note that the reuse, redistribution and reproduction in particular requires that the authors and source are credited.
The license is subject to the *Beilstein Journal of Nanotechnology* terms and conditions: (https://www.beilstein-journals.org/bjnano)

Evaluation of the third sideband

For the sake of simplicity, the bias dependence of the capacitance gradient was neglected in this publication. This additional property could be determined when extending the state observer by an additional state b, which was introduced in Equation 11 of the main manuscript.

Coefficient b is caused by bias-induced band bending and should be observed when semiconducting samples are measured or a semiconducting tip is used as a KFM probe. The effect of band bending can be seen at the third harmonic frequency of the electric modulation, where

$$k_{\mathrm{ts}}^{\mathrm{el}} = k_{\mathrm{ts}}^{\mathrm{el,dc}} + k_{\mathrm{ts}}^{\mathrm{el},\omega} \cos(\omega_{\mathrm{m}} t) + k_{\mathrm{ts}}^{\mathrm{el},2\omega} \cos(2\omega_{\mathrm{m}} t) + k_{\mathrm{ts}}^{\mathrm{el},3\omega} \cos(3\omega_{\mathrm{m}} t), \tag{S1}$$

with

$$k_{\mathrm{ts}}^{\mathrm{el},3\omega} = \frac{1}{8} \frac{\partial^2 \partial C}{\partial z^2 \partial U} U_{\mathrm{ac}}^3. \tag{S2}$$

This result is obtained after considering the Taylor expansion of the capacitance shown in Equation 4 of the main manuscript and applying it to Equation 3.

Parameter b can be determined from the frequency-shift signal using

$$b \equiv \frac{f_0}{4k} \frac{\partial^2 \partial C}{\partial z^2 \partial U} = -\frac{4\, \Delta f_{3\omega_{\mathrm{m}}}}{U_{\mathrm{ac}}^3} \tag{S3}$$

where $\Delta f_{3\omega_{\mathrm{m}}} = -f_0/2k\ k_{\mathrm{ts}}^{\mathrm{el},3\omega}$ is defined as the amplitude of the Δf signal at the third harmonic frequency. This signal was evaluated for the measurement shown in Figure 3 of the main manuscript, where the open-loop TD controller was demonstrated. Figure S1a shows the topography of the sample, Figure S1b shows the error signal obtained from the TD controller after the reconstruction. A slight offset in the error signal, which was expected to be zero-mean, is visible. This offset can

be explained by the presence of band bending, causing an additional frequency component, which was not considered in the measurement model. Figure S1c shows the reconstruction of b using the third harmonic of the electric modulation. The signal is present and the increased value above the graphene flake leads to the increased offset of the error signal at this location.

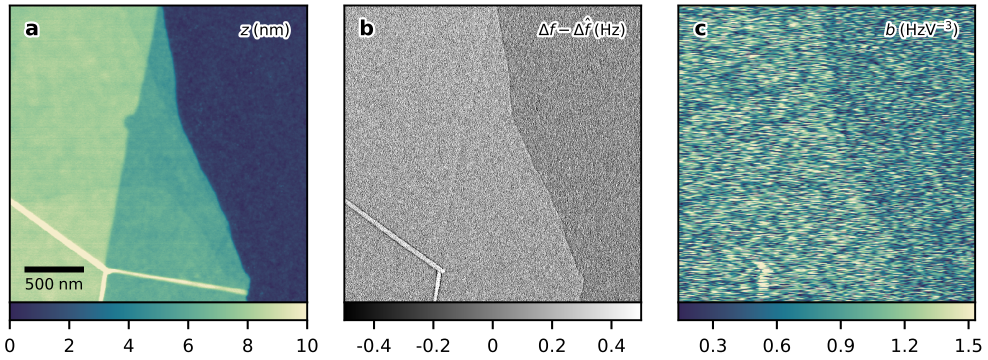

Figure S1: Analysis of bias dependence of the capacitance gradient causing an offset in the error signal of the open-loop TD reconstruction. (a) Surface topography and (b) error signal of the open-loop demonstration. Both figures are reprinted from Figure 3 of the main manuscript. (c) Parameter b, reconstructed from the frequency-shift signal at the third harmonic of the electric modulation.
